# FOXO3a deregulation in uterine smooth muscle tumors

**DOI:** 10.1016/j.clinsp.2024.100350

**Published:** 2024-04-17

**Authors:** Thais Gomes de Almeida, Anamaria Ritti Ricci, Laura Gonzalez dos Anjos, Jose Maria Soares Junior, Gustavo Arantes Rosa Maciel, Edmund Chada Baracat, Katia Candido Carvalho

**Affiliations:** a Laboratório de Ginecologia Estrutural e Molecular (LIM 58), Disciplina de Ginecologia, Departamento de Obstetrícia e Ginecologia, Hospital das Clínicas da Faculdade de Medicina da Universidade de São Paulo (HCFMUSP), São Paulo, SP, Brazil; b Instituto Brasileiro de Controle do Cancer, Mooca, São Paulo, SP, Brazil; c Departamento de Ginecologia Oncológica, Hospital Santa Marcelina, São Paulo, SP, Brazil

**Keywords:** Leiomyoma, Leiomyosarcoma, FOXO3a, Immunohistochemistry, MicroRNA, Gene expression

## Abstract

**Objective:**

The present study aimed to investigate FOXO3a deregulation in Uterine Smooth Muscle Tumors (USMT) and its potential association with cancer development and prognosis.

**Methods:**

The authors analyzed gene and protein expression profiles of FOXO3a in 56 uterine Leiomyosarcomas (LMS), 119 leiomyomas (comprising conventional and unusual leiomyomas), and 20 Myometrium (MM) samples. The authors used techniques such as Immunohistochemistry (IHC), FISH/CISH, and qRT-PCR for the present analyses. Additionally, the authors conducted an in-silico analysis to understand the interaction network involving FOXO3a and its correlated genes.

**Results:**

This investigation revealed distinct expression patterns of the FOXO3a gene and protein, including both normal and phosphorylated forms. Expression levels were notably elevated in LMS, and Unusual Leiomyomas (ULM) compared to conventional Leiomyomas (LM) and Myometrium (MM) samples. This upregulation was significantly associated with metastasis and Overall Survival (OS) in LMS patients. Intriguingly, FOXO3a deregulation did not seem to be influenced by EGF/HER-2 signaling, as there were minimal levels of EGF and VEGF expression detected, and HER-2 and EGFR were negative in the analyzed samples. In the examination of miRNAs, the authors observed upregulation of miR-96-5p and miR-155-5p, which are known negative regulators of FOXO3a, in LMS samples. Conversely, the tumor suppressor miR-let7c-5p was downregulated.

**Conclusions:**

In summary, the outcomes of the present study suggest that the imbalance in FOXO3a within Uterine Smooth Muscle Tumors might arise from both protein phosphorylation and miRNA activity. FOXO3a could emerge as a promising therapeutic target for individuals with Unusual Leiomyomas and Leiomyosarcomas (ULM and LMS), offering novel directions for treatment strategies.

## Highlights

• Higher FOXO3a levels suggest a link to malignancy in Uterine Smooth Muscle Tumors.
• Elevated expression of FOXO3a is connected to LMS prognosis, indicating it is a survival marker.
• MiRNA activity contributes to the imbalance of FOXO3a in Uterine Smooth Muscle Tumors.

## Introduction

Forkhead family of transcription factors – class O (FOXO) transcription factors are involved in several physiological and pathological processes, including aging, stress resistance, neurological diseases, and cancer development.^1^ Within FOXO family, FOXO3a is a crucial protein considered a tumor suppressor by regulating the expression of genes involved in apoptosis, cell cycle arrest, oxidative stress resistance and autophagy.^1^,^2^ Some researchers have suggested that FOXO3a acts as an adaptable player in dynamic homeostasis both in normal and stressed tissue.^2^ In addition, several works have pointed to the relevance of FOXO3a/HER-2 (EGFR) signaling in cancer development and prognosis.^3^ The growth factors ligation to their receptors triggers the signaling cascade that leads to FOXO3a phosphorylation, cytoplasmic translocation, and consequent degradation.^1^,^2^,^4^

Recently, microRNAs (miRNAs) have been described as one of the mechanisms involved in FOXO3a regulation. MiRNAs are non-protein-coding RNA molecules that repress the translation and/or promote mRNA degradation.^5^ The 3′-UTR (3′-Untranslated region) of FOXO3a mRNA contains multiple target sequences for miRNAs. The regulation of FOXO3a by miR-155 has been documented in various cancer types, including breast and lymphoma.^6^,^7^ Additionally, FOXO3a expression is modulated by miR-132, miR-212, and miR-223.Lin and colleagues showed that, in human breast cancer, miR-96 repressing FOXO3a mRNA, leads to expression decreasing in FOXO3a targets (p27 and p21) and increasing of cyclin D1. FOXO3a can also be directly regulated by several other miRNAs in a direct and indirect way.^8^

Usually, FOXO3a loss of function determines deregulation in cell proliferation and DNA damage accumulation, resulting in tissue disorders and several cancer types development (including breast and prostate cancer, glioblastoma, rhabdomyosarcoma and leukemia).^2^,^9^ However, despite the relevance of FOXO proteins in several tumors, very little is known about its role, regulation or expression profile in Uterine Smooth Muscle Tumors (USMTs). These neoplasms can present a broad spectrum of clinical complications from pelvic pain to death.^10^ Among them, Leiomyomas (LM) are the most common benign tumor in reproductive-age women. Despite their indolent clinical behavior, these tumors can induce several troubles both for patients, due to symptoms, and for the government, due the hysterectomies cost by year.^11^ LMS, on the other hand, though rare, is the most common and aggressive USMT. These tumors show high mortality and morbidity rates, with poor response to chemotherapy and radiotherapy.^10^,^12^ Generally, LMS occurs in menopausal women, and metastasis and relapse are very common.^12^ As an intermediary entity, Unusual Leiomyomas (ULM) present features that have similarities with both LM and LMS, encompassing bizarre nuclei leiomyomas, symplastic and pleomorphic leiomyomas, mitotically active, cellular and highly cellular leiomyomas, epithelioid leiomyomas, myxoid leiomyomas, and others.^10^,^13^ They can represent a diagnosis challenge for pathologist concerning their differentiation from LMS.^10^

USMTs characterization by morphologic features and their biologic diversity may be complex mostly due to their wide spectrum of features. The exact origin of those tumors is unknown, and there are still controversies regarding the possibility of LMS arising from a degenerated pre-existing LM or their *de novo* development.^10^,^12^,^13^ The exploration of new markers might help tumors' differential diagnosis, patients’ prognosis, and treatment response prediction, beyond to contribute with potential new targets for specific therapy. Additionally, advances in the knowledge of these neoplasms' biology and behavior will benefit their better clinical management. Here, the authors found FOXO3a with differential gene expression profiles among USMT samples (LM, ULM and LMS), using an array-based gene expression screening analysis of 112 genes well described in the literature associated with several types of cancer. Based on the fact that FOXO3a is described in the literature as a tumor suppressor, the authors decided to assess its expression pattern and role in the USMT. The main focus was to evaluate whether FOXO3a expression profile and regulation could be useful for these tumors diagnosis, prognosis, and treatment prediction.

## Materials and methods

### *Sample selection and classification*

The authors collected and analyzed a total of 55 Leiomyosarcoma (LMS), 103 conventional Leiomyomas (LM), 16 Unusual Leiomyomas (ULM), and 20 Myometrium (MM) samples from frozen and Formalin-Fixed Paraffin-Embedded (FFPE) tissues. These samples were obtained through surgeries conducted between 2000 and 2015. The follow-up period for LMS patients was over thirty months, and relevant clinicopathological data were gathered for each tissue type. The FFPE tissues were sourced from Instituto Brasileiro de Controle do Cancer, Hospital Santa Marcelina, A C Camargo Cancer Center, and Disciplina de Ginecologia do Hospital das Clinicas da Faculdade de Medicina de Sao Paulo. The MM samples were collected from patients who underwent hysterectomies without cancer, inflammatory, or infectious diseases.

The category “Unusual Leiomyoma” (ULM) encompassed tumors that did not fit the criteria for conventional leiomyomas. This included cellular or highly cellular, atypical, metastatic, and mitotically active leiomyomas, as well as STUMPs, forming an intermediate group for comparative analysis.^10^,^13^

Medical records of participants were examined, gathering information such as age, primary complaints, associated secondary diseases, surgical procedures, treatments, tumor recurrence, and metastasis. Tumor staging followed the FIGO-2009 guidelines,^14^ while histological grading was based on nuclear polymorphism and mitotic index. The study received ethical approval from the Research Ethics Committee of Faculdade de Medicina da Universidade de São Paulo-FMUSP and the Research Ethics Committee of Hospital das Clinicas da Faculdade de Medicina da Universidade de São Paulo ‒ CAPpesq (Nos. 143/11 and 0845/11) and was conducted in accordance with the Helsinki Declaration.

### *RNA extraction and qRT-PCR for gene and miRNAS expression analysis*

Total RNA was isolated from FFPE samples (5 MM, 5 LM, 8 ULM, and 37 LMS) following established protocols.^15^,^16^ Gene expression analysis focused on 112 genes spanning diverse biological pathways, with exclusive consideration of genes linked to FOXO3a regulation. For cDNA synthesis, High-Capacity kits (Applied Biosystems, USA) were utilized, employing 2 µg of total RNA. qRT-PCR reactions, executed in duplicate, utilized 1.2 µL cDNA and 3.8 µL TaqMan Gene Expression Master Mix (Applied Biosystems, USA), conducted on the QuantStudio 12K Flex Open Array Real-Time PCR System (Applied Biosystems, USA). Cycling conditions adhered to manufacturer recommendations. Housekeeping genes included ACTB, B2M, GAPDH, GUSB, HPRT1, and RPLP0. Data analysis employed expression suite software v1.0.3 employing the comparative Cτ (ΔΔCτ) method (Life Technologies, USA), using MM samples as references.

For miRNA expression analysis, tissues were dissected to optimize tumor cell yield. Qiagen miRNeasy FFPE Kit facilitated total RNA extraction from tissues. For cDNA synthesis and relative miRNA quantification, Qiagen miScript II RT Kit, miScript SYBR Green PCR Kit, and miScript miRNA PCR Arrays were employed. Specific miRNA sequences were identified using the MIHS 102Z cancer-related development miRNA PCR Array (Qiagen, Hilden, Germany). Reactions and analyses followed established protocols.^15^,^16^

### *Immunohistochemistry (IHC) and protein expression quantification*

All cases were reviewed and selected based on the evaluations of two independent pathologists (IWC and FAS). Discordant cases were evaluated by another observer and retrieved for discussion and consensus. The TMA blocks, hematoxylin and eosin, and immunohistochemical analysis were performed as previously described.^17^

All IHC reactions for FOXO3a (1:100, pressure cooker, pH6.0, rabbit polyclonal antibody, Novus Biological Inc, USA), FOXO3a-Phospho Ser-253 (1:100, pressure cooker, pH6.0, rabbit polyclonal antibody, Arigobio, USA), EGF (1:50, pH6.0, mouse monoclonal antibody, pressure cooker + enzymatic digestion, DakoCytomation, USA), VEGF (1:100, pressure cooker, ph6.0, rabbit polyclonal antibody, Abcam) and HER-2 (1:2,000, pressure cooker, pH6.0, rabbit polyclonal antibody, A0485, DakoCytomation, USA) detection were standardized on conventional slides before the analysis was performed on the TMA. Negative controls were obtained by omitting the primary antibody or including nonreactive IgG. All IHC reactions were performed in duplicate.

For visual evaluation, each spot was scored for staining intensity and the positive cell quantitation (frequency). To determine the protein immunostaining score, the authors used a design proposed elsewhere for nuclear and cytoplasmic protein staining.^17^,^18^ HER-2 protein evaluation was performed using the internationally recognized scoring system from the ASCO/CAP guideline.^19^

### *Fluorescent and colorimetric in situ hybridization (FISH and CISH) for HER-2 gene assessment*

Vysis LSI Dual Color HER-2/CEN17 (Abbott Laboratories, Abbott Park, IL, USA) was used for FISH analysis. All the pretreatment phase was performed in a semi-automated machine VP2000 Processor™ (Abbott). Reactions were performed as described previously.^24^ FISH slides were analyzed by observing the presence of green signals (for HER-2 gene) and red signals (chromosome 17). The same proportion of both green and red signals indicate no copy number alterations; proportion ≥2.5 (2;10) could be considered gene amplification. Slides were evaluated by pathologists using fluorescent microscopy.

Automated SISH was performed on Ventana Benchmark XT (Ventana Medical Systems, Tucson, AZ, USA) using whole sample tissues´. Ultraview Inform HER-2 DNA probe and Inform Chromosome 17 centromere (cen17) probe were visualized on the same slide. Assay conditions were modified to obtain optimal results. The protocol (deparaffinization, pretreatment, hybridization, stringency wash, signal detection and counterstaining) was fully automated. HER-2 probe was denatured at 95°C for 20 min and hybridized at 52°C for 6h. The chromosome 17 centromere probe was denatured at 95°C for 20 min and hybridized at 44°C for 6h. Stringency washes were performed at 72°C for 8 min. The silver signal for HER2 was revealed by sequential silver reactions. The signal of the centromere was visualized with the RedISH Naphtol reaction. The tissues were counterstained with Hematoxylin II and Bluing Reagent.^17^

### *Western blot assays for antibody specificity assessment*

Breast cancer (MCF7, ATCC) cells were used for antibody specificity analysis by western blot. Cell lines were grown in a specific medium indicated by ATCC, supplemented with 10% fetal bovine serum and 0.1% penicillin-streptomycin. After the removal of the supernatant, the cells were washed and scraped into Phosphate-Buffered Saline (PBS 1X). The total protein extract from all samples was obtained with RIPA buffer (50 mM Tris–HCl Ph 8.0, 150 mM NaCl, 1% NP-40, 0.5% sodium deoxycholate, and 0.1% SDS). Protein concentration was determined using a Brad-ford assay and 20‒30 μg of protein were separated using 10% SDS-polyacrylamide gels. After protein transference to nitrocellulose or PVDF membranes (Thermo Fisher Scientific, Waltham, MA, USA), monoclonal antibodies against FOXO3a total e ‒phospho (1:1000), in PBS-Tween (0,001%) containing 5% non-fat milk, was added to the membrane at 4°C overnight. Actin-b was included as a positive control of the protein detection in all the experiments.

### *Statistical analysis*

Fisher's exact test was used to evaluate associations between protein expression and clinicopathological parameters. Overall and disease-free survival rates were calculated using the Kaplan-Meier method based on a follow-up of 5 years for all ULM and LMS patients included in this study. The odds ratio was calculated using the log-rank test. Hazard Ratio (HR) and their 95% (95% IC) interval of confidence were calculated using the regression model of Cox.

All calculations were performed using GraphPad Prism 5.0 statistical software (San Diego, CA, USA) and SPSS v.25 for Windows (SPSS Inc. Chicago. IL, USA). Statistical significance was accepted for p-values ≤0.05.

## Results

The average age of Leiomyoma (LM), Unusual Leiomyoma (ULM), and Leiomyosarcoma (LMS) patients was 44 ± 7.0, 43 ± 9.0, and 55.4 ± 15.2 years old, respectively. Among LM patients, 59% were Caucasian, while 44% of ULM patients were Caucasian. In the LMS group, 59% were Caucasian, 13% were non-Caucasian, and 5% were Asian. Menopausal status was reported as 0%, 6%, and 46% in LM, ULM, and LMS cases, respectively. Recurrence was observed in 18% of ULM patients, whereas LMS had a recurrent/relapsed rate of 81.8%. Among LMS patients, 76.4% presented a high-grade lesion, with the primary symptom being vaginal bleeding (43.6%). It is noteworthy that 25.5% of LMS patients did not use contraceptives, 27.3% underwent replacement therapy, and 25.5% were multiparous.

Forty percent of these women did not perform treatment, 29.1% were submitted to chemotherapy, and 20% performed radiotherapy. All additional clinical and pathological data from LMS and LM patients are presented in Table 1 and Supplementary Table 1, respectively.

**Table 1:** Clinical and pathological features of LMS patients (n=55).

**Feature**		**n=55**
Age (Diagnosis)	Mean (SD)	55.4 (15.2)
	Median (min‒max value)	52 (27–91)
	≤50 years	25 (45.5%)
	>50 years	30 (54.5%)
Histological grade	Low	13 (23.6%)
	High	42 (76.4%)
Mainly Symptom	Vaginal bleeding	24 (43.6%)
	Pelvic pain	22 (40%)
	N.A.	9 (16.4%)
Adjuvant Treatment	No	22 (40.0%)
	Chemotherapy	16 (29.1%)
	Radiothetapy	11 (20.0%)
	Both	6 (10.9%)
Metastasis	No	22 (40%)
	Yes Local	9 (16.4%)
	Distance	24 (43.6%)
Recurrence	No	10 (18.2%)
	Yes	45 (81.8%)
Figo Stage	I	20 (36.4%)
	II	10 (18.2%)
	III	8 (14.5%)
	IV	17 (30.9%)
Disease Stage	Uterine disease (including uterine cervix)	18 (32.7%)
	Pelvic involvement	10 (18.2%)
	Extra pelvic disease (positives lymphnodes)	8 (14.5%)
	Metastasis (liver and lungs)	16 (29.1%)
	N.A.	3 (5.5%)

*HRT, Hormonal Reposition Treatment; SD, Standart Deviation; min, Minimum value; max, Maximum value.

**Figure 1:**
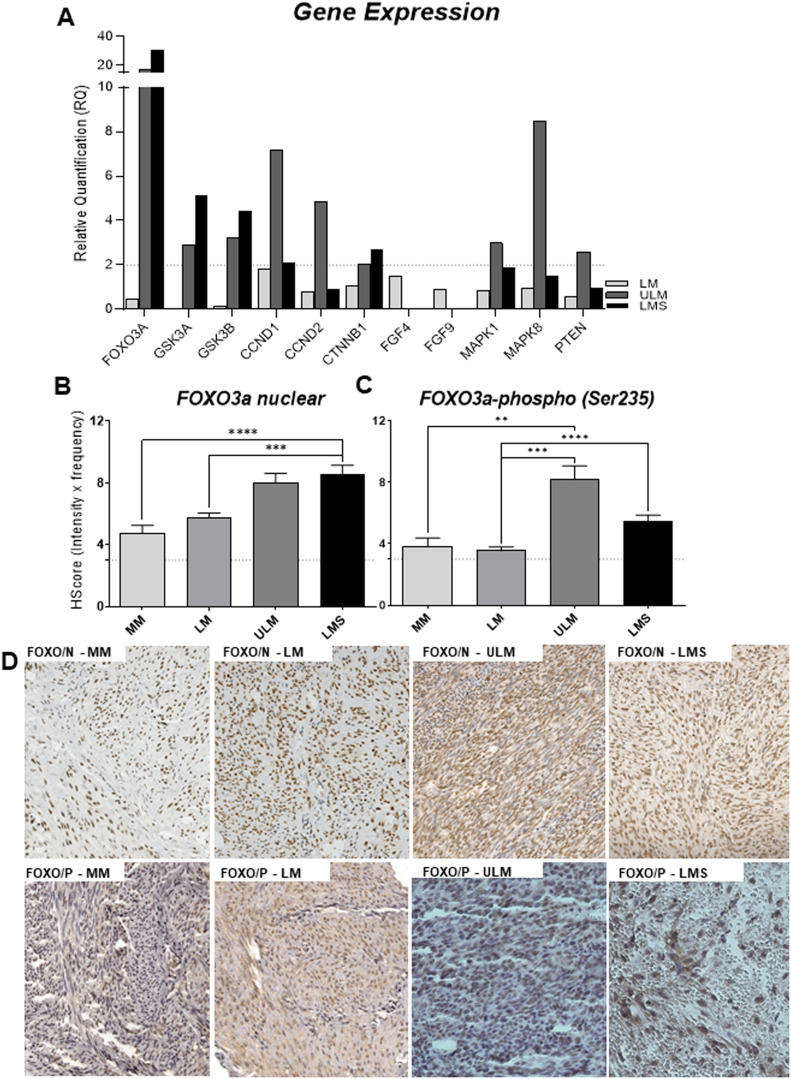
FOXO3a expression profile in USMT patients ´samples. (A) Gene expression of FOXO3a and genes related to its regulation. The analysis was performed using a Taqman PCR array-based screening, including 112 genes previously described in several cancer types. The relative expression values (RQ) were obtained using as reference control a pool of myometrium samples. (B) Semi-quantitative analyses of FOXO3a nuclear (wild type) protein expression in the samples. (C) Semi-quantitative analyses of FOXO3a cytoplasmic (phosphorylated form) protein expression. (D) Representative photomicrographs of protein expression profile in the USMT samples (original resolution 40×). FOXO/P, Phosphorylated Protein (cytoplasmic); FOXO/N (nuclear) – total protein; MM, Myometrium, LM, Leiomyoma, ULM, Unconventional Leiomyoma; LMS, Leiomyosarcoma. Dashed line indicates the cut off value for positive protein expression (≥3). Reference value of expression is indicated by doted arrow (=1); p-values are indicated.

Samples underwent dual pathology review, with representative Hematoxylin-Eosin photomicrographs in Figure S1. An initial gene expression screening encompassing 112 literature-linked genes from pivotal cellular signaling pathways related to diverse cancer types was conducted (Supplementary Table 2). qRT-PCR highlighted FOXO3a and 11 pathway-related genes exhibiting substantial differential expression in USMT samples (Fig. 1A). FOXO3a expression was notably elevated, approximately 30-fold (RQ) in LMS samples and 18-fold in ULM, compared to LM and MM samples. GSK3α, GSK3β, CCND1, and CTNNB1 also displayed elevated expression in ULM (RQ = 2.89, 3.23, 7.17, and 2.01, respectively) and LMS (RQ = 5.11, 4.42, 2.08, and 2.72, respectively). ULM samples exhibited increased expression of CCND2, MAPK1 (ERK1), MAPK8 (JNK1), and PTEN (Fig. 1A).

FOXO3a overexpression was corroborated at protein levels via Immunohistochemical (IHC) analysis across patients' samples on Tissue Microarray (TMA) slides. Both nuclear and phosphorylated (Ser253) cytoplasmic forms of FOXO3a were assessed (Fig. 1 B‒D). For nuclear FOXO3a expression (wild-type protein), over 80% of LMS samples exhibited positive protein expression, while ULM showed 36.4% strong/weak expression and 27% moderate staining. LM samples had 25% strong and 31% moderate expression. Comparatively, ULM and LMS had higher protein expression scores, with statistical disparities between LMS and both LM and MM (Fig. 1B). Similarly, phosphorylated (Ser253) FOXO3a (cytoplasmic) was elevated in ULM and LMS, contrasting with MM and LM samples (Fig. 1C). ULM demonstrated the highest protein levels. Figure 1D shows the staining pattern observed for nuclear cytoplasmic forms of the FOXO3a protein.

Forty-eight (out of 56) samples of LMS were evaluated for nuclear FOXO3a and 47 were assessed for its phosphorylated form. The protein levels were considered negative when the Hscore values were < 3 and positive for Hscore >3. No significant correlation was observed between patients clinical and pathological features and nuclear protein expression (p > 0.05), but metastasis occurrence (p = 0.035) was associated with cytoplasmic FOXO3a expression (Table 2). A marginal significance was found for relapse (p = 0.078) and metastasis site (p = 0.061) with phosphorylated protein expression in LMS samples (Table 2).

**Table 2:** FOXO3a protein expression and patients’ features association analysis.

**Characteristics/Category**	**FOXO N**		**p-value**	**FOXOp**		**p-value**
	**Negative**	**Positive**		**Negative**	**Positive**	
	**n=9 (%)**	**n=39 (%)**		**n =11 (%)**	**n =36 (%)**	
Age (Diagnosis)			0.140^a^			0.505^a^
≤50 years	2 (8.7)	21 (91.3)		6 (28.6)	15 (71.4)	
>50 years	7 (28.0)	18 (72.0)		5 (19.2)	21 (80.8)	
Mainly Symptom			0.695^a^			0.243^a^
Vaginal bleeding	5 (25.0)	15 (75.0)		7 (36.8)	12 (63.2)	
Pelvic pain	3 (15.8)	16 (84.2)		4 (20.0)	16 (80.0)	
Adjuvant treatment			0.653^a^			0.662^a^
No	2 (11.1)	16 (88.9)		4 (21.1)	15 (78.9)	
Chemotherapy	3 (20.0)	12 (80.0)		3 (23.1)	10 (76.9)	
Radiotherapy	3 (30.0)	7 (70.0)		4 (36.4)	7 (63.6)	
Both	1 (20.0)	4 (80.0)		0	4 (100)	
Disease staging			0.213^a^			0.700^a^
Uterine disease (including uterine cevix)	3 (18.8)	13 (81.3)		4 (23.5)	13 (76.5)	
Pelvic involvement	0	10 (100)		4 (40.0)	6 (60.0)	
Extra pelvic disease (positives limphnodes)	3 (37.5)	5 (62.5)		1 (20.0)	4 (80.0)	
Metastasis (liver and lungs)	3 (23.1)	10 (76.9)		2 (16.7)	10 (83.3)	
Relapse ocurrence			0.613^a^			0.078^a^
No	2 (25.0)	6 (75.0)		4 (50.0)	4 (50.0)	
Yes	4 (12.9)	27 (87.1)		5 (17.2)	24 (82.8)	
Relapse site			0.583^a^			0.400^a^
Absent	2 (25.0)	6 (75.0)		4 (50.0)	4 (50.0)	
Local	1 (11.1)	8 (88.9)		1 (16.7)	5 (83.3)	
Distant				4 (17.4)	19 (82.6)	
Metastasis ocurrence			1^a^			0.035^a^
No	1 (25.0)	3 (75.0)		3 (75.0)	1 (25.0)	
Yes	8 (18.2)	36 (81.8)		8 (18.6)	35 (81.4)	
Metastasis site			0.506^a^			0.061^a^
Absent	1 (25.0)	3 (75.0)		3 (75.0)	1 (25.0)	
Local	4 (28.6)	10 (71.4)		4 (26.7)	11 (73.3)	
Distant	4 (14.8)	23 (85.2)		4 (14.8)	23 (85.2)	
Both	0	3 (100)		0	1 (100)	

SD, Standart Deviation; min, Minimum value; max, Maximum value.

a Fisher Test.

**Figure 2:**
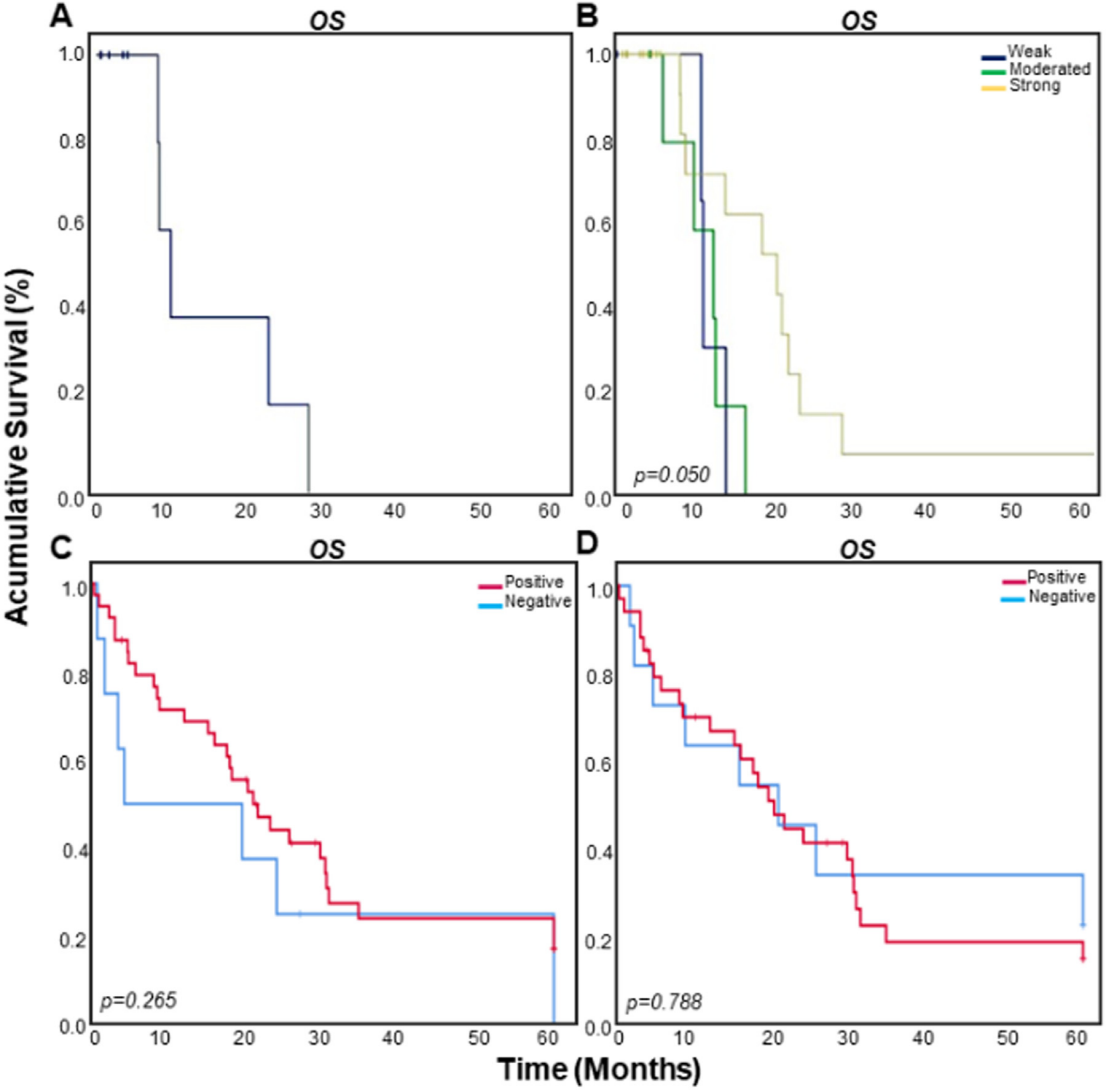
Kaplan-Meier curves showing the overall and disease-free survival rates (months) of LMS patients. (A) Overall Survival (OS) of all the LMS patients included in the present study. (B) OS of the patients according to total Foxo3a protein expression divided into 3 categories (weak, moderated, and strong protein expression). (C) OS of the patients according to nuclear (wild type) FOXO3a. (D) OS of the patients according to cytoplasmic (phosphorylated form) of FOXO3a. The data are presented as percentage (%) and the time was plotted in months.

Overall Survival (OS) and Disease-Free Survival (DFS) were analyzed considering the time between the diagnosis and decease, or the last information (relapse, recurrence, and metastasis). Follow-up time was established as 60 months and live patients (for OS) or misses of view (both to OS and DFS) were included in the censored group. Figure 2A presents the OS curve observed for all the LMS patients. FOXO3a strong protein expression (weakly, moderate and strong staining), was associated with higher OS time (Fig. 2B). The authors also evaluated positive or negative categories of protein the expression, considering its cell location separately (nuclear or cytoplasmic) and, although a more precocious curve of death was obtained for patients with negative nuclear FOXO3a expression, no significant differences were found (Figs. 2 C and D).

The Hazard Ratio (HR) values were assessed using the regression model of Cox to analyze the prognosis value of FOXO3a expression (Table 3). The global group survival probability is presented according to the features. Adjuvant therapy (p = 0.015), disease staging (p = 0.021), and relapse (p = 0.008) showed significant association with overall survival in LMS patients. Metastasis occurrence and age showed marginal values of significance concerning overall survival (p = 0.074 and 0.071, respectively), and five years survival was 15.5% (Table 3). Although no statistical significance was observed, a higher percentage of deaths occurred in patients with negative FOXO3a nuclear expression.

**Table 3:** Overal survival of LMS patients.

**Characteristics**	**Total of deaths**	**HR (95% CI)**	**p-value**	**Survival % (5 years)**
Geral	40/54			15.5%
FOXO N				
Negative	7/8	1.00		0
Positive	29/39	0.63 (0.28‒1.44)	*0.275*	17.1%
FOXO p				
Negative	8/11	1.00		22.7%
Positive	26/35	1.11 (0.50‒2.47)	*0.790*	15.1%
Age group				
≤50 years	16/24	1.00		24.6%
>50 years	24/30	1.78 (0.95‒3.36)	*0.074*	8.5%
Mainly Symptom				
Vaginal bleeding	16/23	1.00		8.2%
Pelvic pain	15/22	0.89 (0.44‒1.80)	*0.742*	28.3%
Adjuvant treatment				
No	14/22	1.00		24.9%
Chemotherapy	16/16	2.47 (1.19‒5.14)	*0.015*	0
Radiotherapy	7/10	1.30 (0.52‒3.23)	*0.576*	22.9%
Both	3/6	0.94 (0.27‒3.34)	*0.924*	40.0%
Disease staging				
Uterine disease (including uterine cevix)	11/17	1.00		17.7%
Pelvic involvement	7/10	1.28 (0.50‒3.31)	*0.610*	30.0%
Extra pelvic disease (positives limphnodes)	7/8	1.51 (0.58‒3.92)	*0.397*	0
Metastasis (liver and lungs)	14/16	2.56 (1.15‒5.72)	*0.021*	7.0%
Relapse				
No	1/8	1.00		80.0%
Yes	28/34	15 (2.02‒111.4)	*0.008*	7.5%
Metastasis ocurrence				
No	1/3	1.00		66.7%
Yes	39/51	6.3 (0.85‒46.6)	*0.071*	11.6%

**Figure 3:**
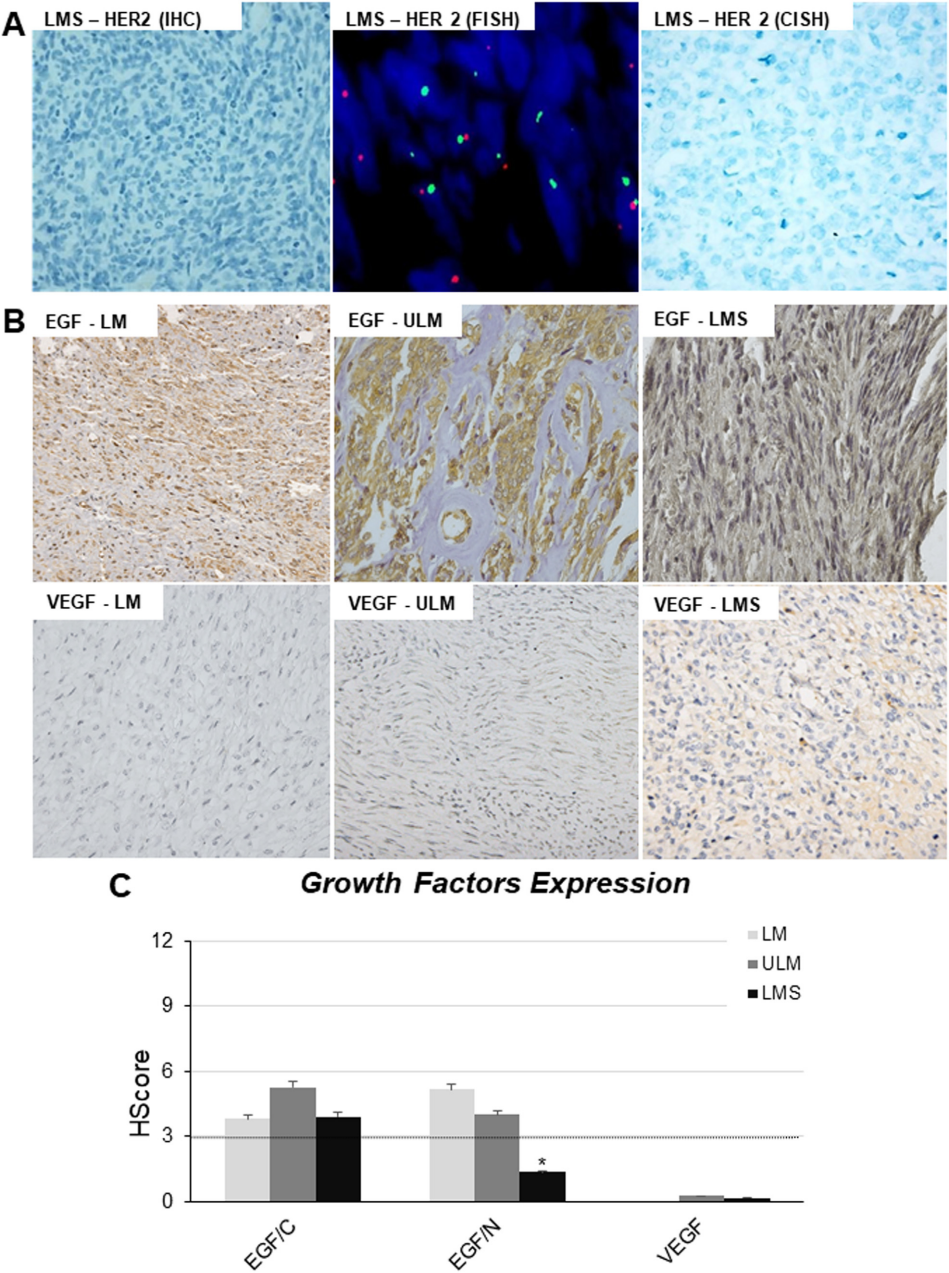
HER-2/EGF pathways members’ expression in USMT. Photomicrographs show in panel A, representative samples evaluated for HER-2 (IHC, FISH and CISH) detection (original resolution 40×). (B) Representative photomicrographs of the samples stained for EGF and VEGF detection by IHC (original resolution 40×). (C) Semi quantitative analyses of EGF (EGF/C ‒ Cytoplasmic and EGF/N ‒ Nuclear) and VEGF protein expression. Dashed line indicates the cut off value for positive protein expression (≥3). MM, Myometrium, LM, Leiomyoma; ULM, Unconventional Leiomyoma; LMS, Leiomyosarcoma.

**Figure 4:**
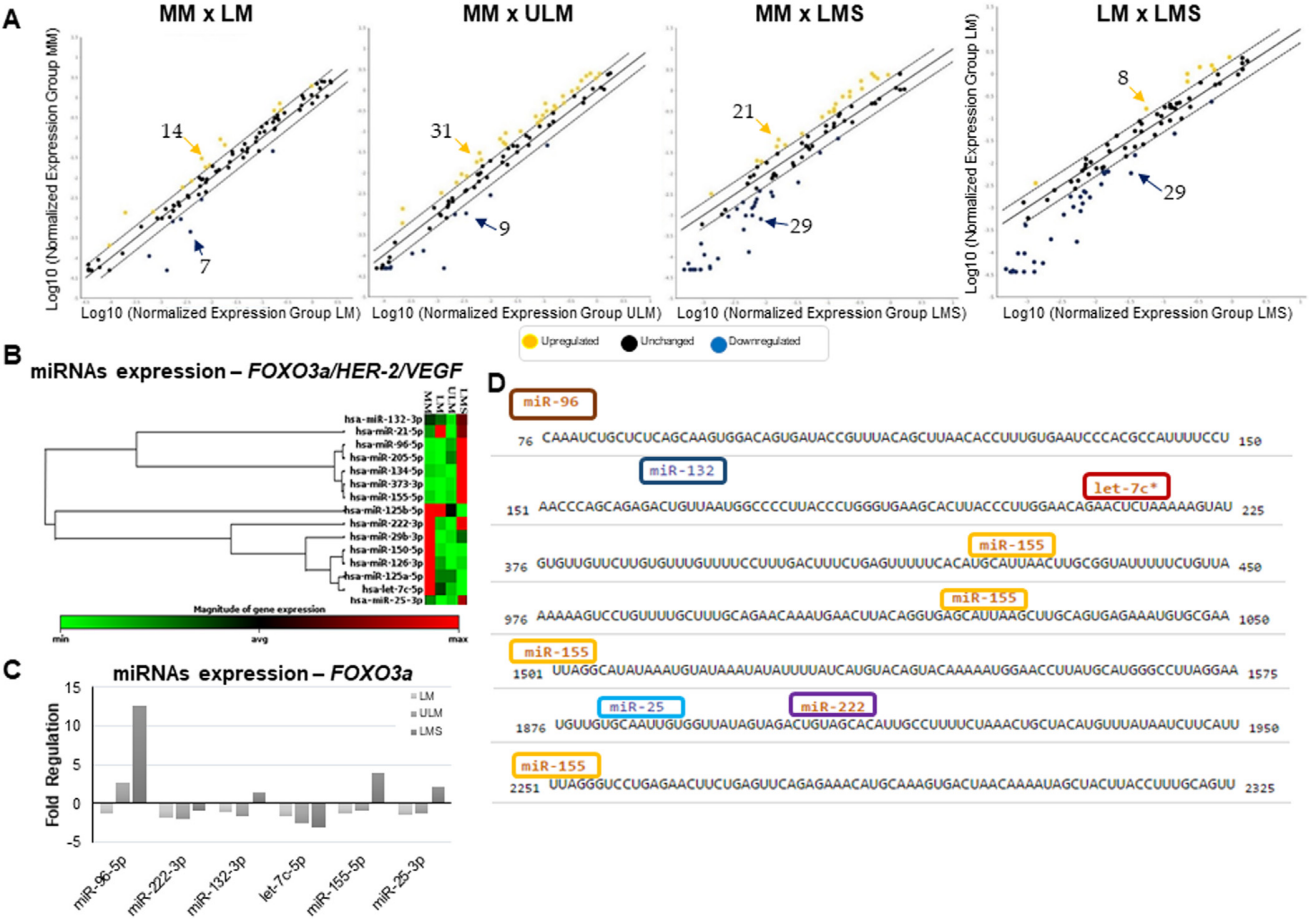
miRNAs expression in USMT samples. (A) Scatter Plots comparing all the 84 oncomirs evaluated by qRT-PCR in the tumors and control samples (MM). (B) Heat map showing the unsupervised expression profile of the 15 miRNAs sequences found as regulators of HER-2, EGF and VEGF, included in the array platform. (C) Validation of the expression of FOXO3a regulators miRNAs. MM pool of samples were used as references for gene expression. (D) Predicted microRNA targets and target downregulation scores (microRNA.org ‒ Targets and Expression). MM, Myometrium; LM, Leiomyoma; ULM, Leiomyoma; LMS, Leiomyosarcoma.

Subsequent analyses encompassed the assessment of HER-2 and EGFR membrane receptor expression to investigate the potential involvement of the EGF pathway in FOXO3a deregulation (Fig. 3). However, no positive staining was observed for both receptors in the samples. Given the frequent association of negative HER-2 expression with gene alterations, FISH (on TMA slides) and CISH (on whole LMS tissues) were conducted to evaluate the gene status. Figure 4A illustrates the reaction profiles for HER-2 detection using IHC, FISH, and CISH. Neither FISH nor CISH reactions showed chromosomal alterations in the HER-2 gene. Additionally, the authors examined EGF and VEGF as ligands in the pathway potentially responsible for FOXO3a phosphorylation and subsequent inactivation. EGF exhibited both cytoplasmic and nuclear expression, while VEGF was exclusively observed in the cytoplasmic compartment (Figs. 3 B and C). Immunohistochemistry revealed lower levels of these proteins; however, considering only positive samples, higher cytoplasmic EGF expression was observed in ULM compared to LMS and LM (Fig. 3C). Nuclear EGF expression was lower in LMS samples (Fig. 3 B and C). VEGF expression was similar in ULM and LMS samples, with notably lower values (below the cutoff = 3) and negative expression in LM samples (Fig. 3C). Due to the limited number of positive samples, statistical analysis could not be conducted.

Another significant regulatory mechanism considered in gene expression is miRNAs. These molecules are known to control HER-2, growth factors, and FOXO3a expression. Evaluating their profiles could aid in understanding the gene and protein expression results for USMT samples. The authors assessed 84 miRNAs implicated in the development of several types of cancer and potentially involved in those three genes regulation (Fig. 4). The authors used MM samples as a reference for miRNA expression in normal uterine tissue due to its mesenchymal nature and similar location and biological changes to benign (LM and ULM) and malignant mesenchymal tumors (LMS).

Initial miRNA expression analysis focused on comparing tumor samples to normal tissue (Fig. 4A). Between MM and LM samples, 21 miRNAs showed differential expression (14 upregulated and 7 downregulated in LM). For MM vs. ULM, 31 miRNAs were upregulated and 9 downregulated in tumors. The MM vs. LMS comparison revealed 50 miRNAs with distinct expression (21 upregulated and 29 downregulated in LMS). Specifically considering miRNA regulators of HER-2, VEGF, EGF, and FOXO3a from literature and the arrays, 15 sequences displayed differential expression, linked with FOXO3a/VEGF/HER-2 deregulation. The clustergram (Fig. 4B) displays expression values across samples for each tissue type, utilizing a color scale. Tumors, particularly LMS, displayed notably different profiles from normal tissue (reference group ‒ MM). Among FOXO3a regulators, six miRNAs were identified (miR-96-5p, miR-222-3p, miR-132-3p, let7c-5p, miR-155-5p, and miR-25-3p). For HER-2, absent in 100% of samples without deletion/loss of the encoding gene, four regulatory miRNAs (miR-125b-5p, miR-21-5p, miR-125a-5p, and miR-205-5p) showed differential expression. Concerning VEGF regulatory miRNAs, five exhibited distinct regulation in the present study's samples (miR-134-5p, miR-373-3p, miR-29b-3p, miR-150-5p, and miR-126-3p). No differentially expressed miRNA involved in EGF regulation was found in the array-based analysis.

The expression of the six miRNAs identified as FOXO3a regulators (miR-96-5p, miR-222-3p, miR-132-3p, let7c-5p, miR-155-5p, and miR-25-3p) was validated using a more sensitive and specific qRT-PCR detection method (Taqman® probes and primers, Thermo Fisher Scientific). Figure 4C displays miRNA-96-5p and miR-155-5p as the most expressed in LMS samples, followed by miR-25-3p and miR-132-3p, with let-7c-5p having the lowest expression. miR-155-5p appears to be a significant miRNA regulator of FOXO3a, as the authors found four binding sites described for this miRNA in the gene sequence (Fig. 4D).

**Figure 5:**
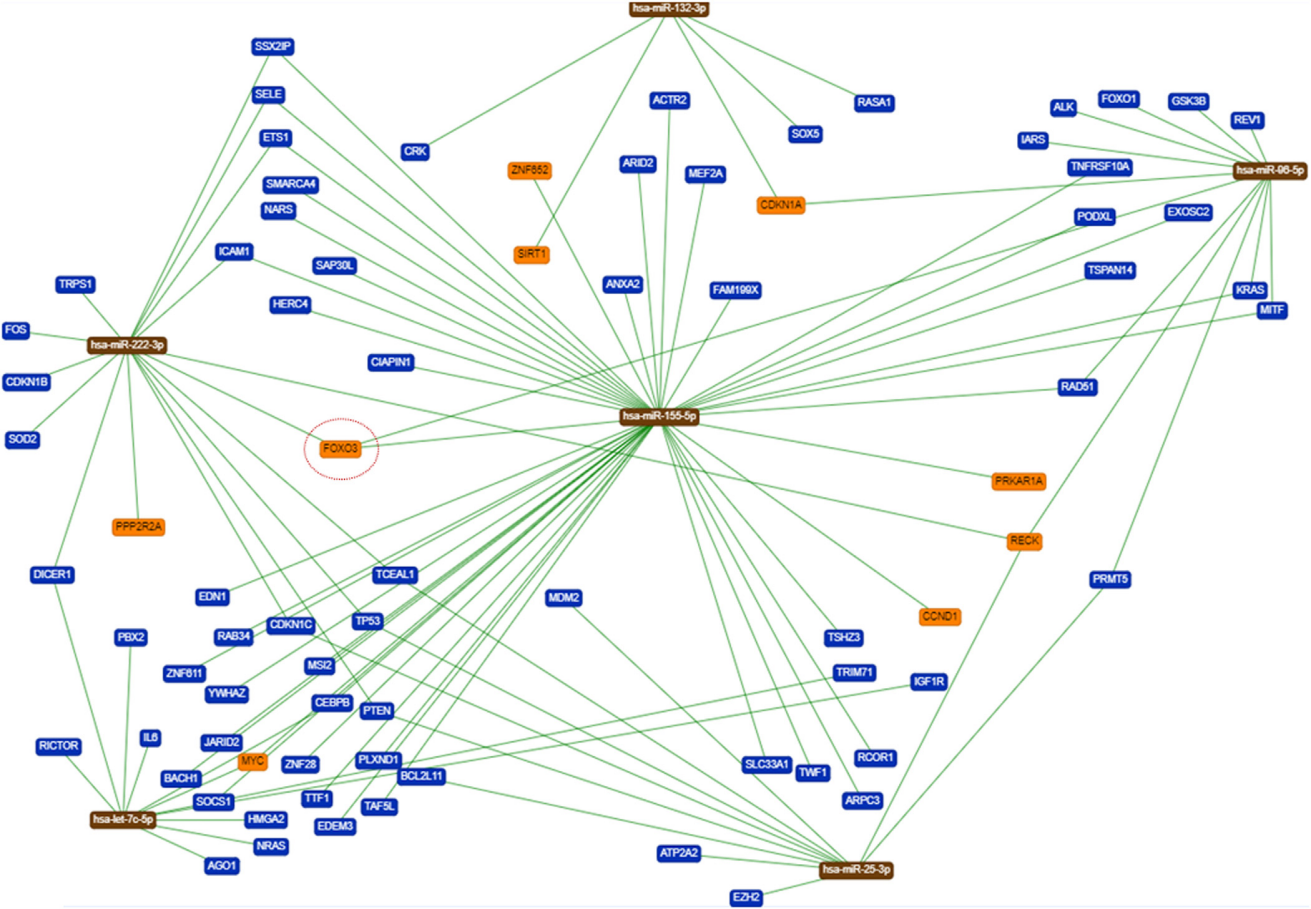
Interaction network of miR-96-5p, miR-222-3p, miR-132-3p, let7c-5p, miR-155-5p and miR-25-3p and their main target-genes. The network includes only regulation with evidence defined in the literature. FOXO3a is indicated by the red dotted circle. The green lines indicate the interaction among the miRNAs and their target genes. The red dotted circle indicates FOXO3a as the focus of the present study.

Moreover, an in-silico assessment of FOXO3a gene interactions with its associated miRNAs and genes elucidated pertinent signaling pathways linked to FOXO3a expression. miRTARBase exclusively incorporated validated outcomes for FOXO3a and regulatory miRNAs, substantiated via gene reporter assays, western blots, and qRT-PCR. Each miRNA exhibited potential to modulate numerous genes, some of which significantly influence FOXO3a regulation or might be impacted by its expression (Fig. 5). The robust green connections directly tied to FOXO3a signify miRNAs wielding substantial influence (miR-96, 155, and 222) on gene regulation. While in silico analysis didn't unveil a direct robust correlation between let7c-5p and FOXO3a, a negative correlation surfaced in their expression across IHQ (-0.002887213) and qRT-PCR (-0.00752953) assays in LMS samples (data not shown).

**Figure 6:**
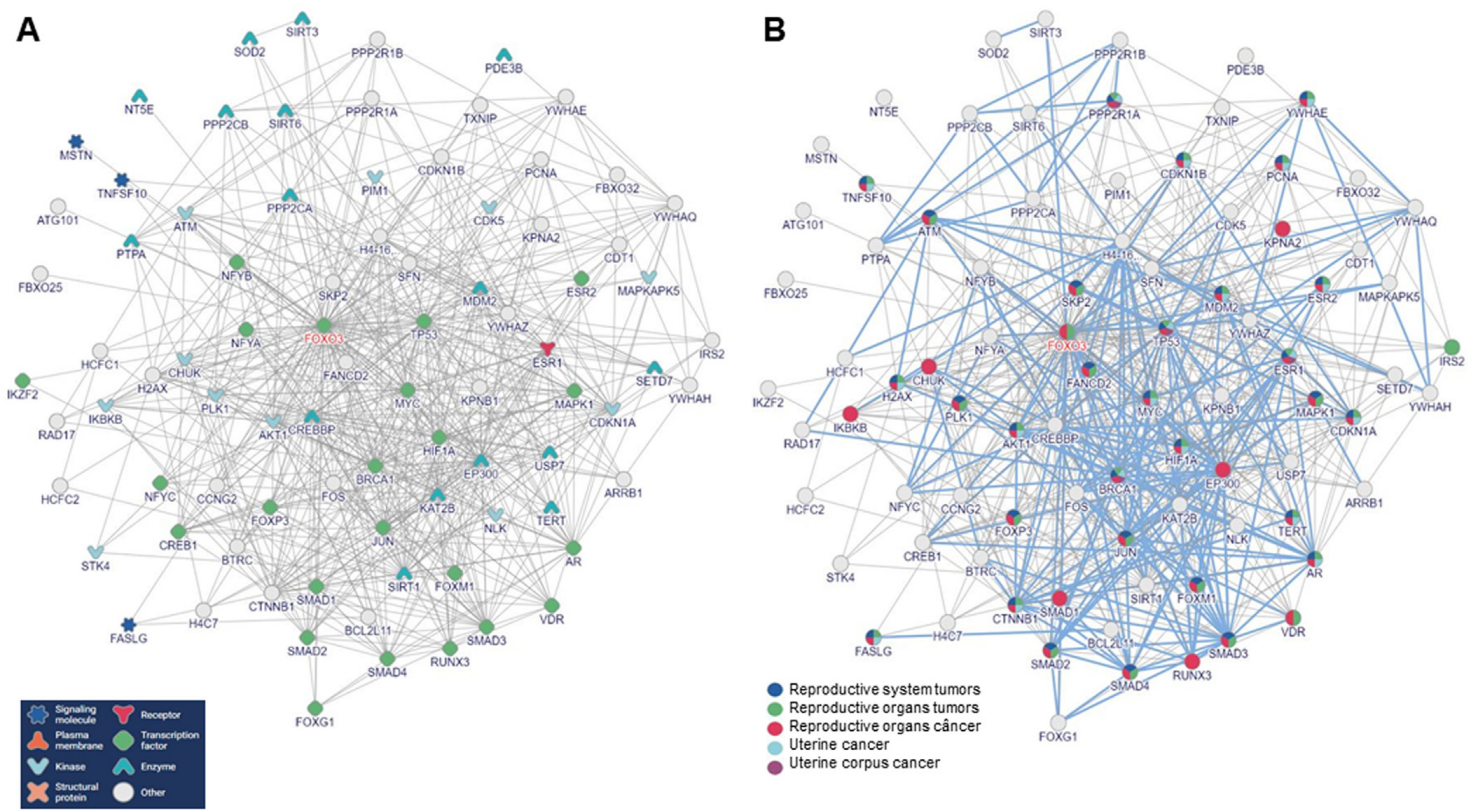
Interaction network of FOXO3A and related genes described as associated with gynecological tumors. (A) Genes biological function in the cells. (B) Female tumors described in the literature as associated with the signaling of the genes included in the network (p<0.0001).

Furthermore, supplementary network analysis (Fig. 6) spotlighted multiple genes ensconced within pivotal biological pathways entwined with tumor development, cell migration, invasion, metastasis, and patient prognosis. Figure 6 comprehensively illustrates all genes co-expressed with FOXO3a in gynecological tumors as substantiated by literature. The biological functions of these genes (Fig. 6A) and their delineated biological process pathways (blue lines) pertinent to gynecological tumors (Fig. 6B) are also delineated.

## Discussion

As a heterogeneous group of tumors, USMT ranges from benign to aggressive malignant tumors (LM and LMS, respectively).^10^, ^11^, ^12^ Here, the authors included LM, LMS, and Unconventional Leiomyoma (ULM) in the present analyses, focusing on their morphological and clinical features. The goal was to compare these tumors' gene expression profiles and to identify potential new markers for their differential diagnosis, prognostic, and treatment prediction. Initially, the clinical and pathological data of the patients were evaluated. The average age of LMS women was 55.4 years old, corroborating the literature[12] as well as observed for LM and LMU. The treatment of choice for LMS patients was hysterectomy (98%), which is considered the gold standard treatment for all uterine sarcomas.^12^,^20^ Occurrence of metastasis was observed in 59% of the cases, the lungs being the main affected site. The high tendency of recurrence and distant metastasis has already been well documented for this kind of tumor.^12^ Overall Survival (OS) at two years is generally less than 50%, with hematogenous metastasis, mainly to the lungs.^17^ The present results showed lower than 30% of OS after 30 months of initial diagnosis, and DFS of 0% at this same time. These data corroborate the higher rates of recurrence and aggressiveness of LMS described in the literature.^20^ As expected, OS was lower in patients with relapse and metastasis occurrence as well as lung metastatic disease. The mean follow-up of the patients was 36 months.

The molecular study of the events involved in the development of different types of cancer has led to new strategies used in their diagnosis and treatment. The origin of the USMT is still not well understood alike there are still not effective and specific therapies for these tumors. Molecularly, uterine Leiomyoma (LM) presents important genetic dysfunctions such as alterations in RAD51, BRCA1, MED12 and HMGA2,^21^ genes related to DNA repair and cell growth. This tumor presents biological and morphological characteristics like uterine Leiomyosarcomas (LMS), making it difficult to establish a differential diagnosis. LMS, in turn, shows molecular alterations in genes responsible for several functions such as p51, p16, and BCL-2, responsible for regulating the cell cycle, growth and apoptosis, in addition to the downregulation of tumor suppressors’ genes such as RB1, DCC, NM23, WT1, D14S267, P16 and PTCH.^22^ The studied group has performed several molecular analyses in USMT patients and samples.^15^,^16^,^23^,^24^ The present study was started after qRT-PCR data showed FOXO3a with significant differential levels of expression among USMT samples. Initially, an array-based method showed FOXO3a with an increasing expression profile in LM, ULM and LMS, compared to normal Myometrium (MM). Gene expression data was validated by IHC analyses both to wild-type protein as well as to the phosphorylated one. The loss of FOXO3a expression has been associated with poor prognosis in several types of cancer.^2^,^9^ In ovarian cancer, the loss of FOXO3a function may limit the sensitivity of cancerous cells to chemotherapy.^25^ Some chemotherapeutic drugs, currently used in the treatment of breast cancer and in acute myeloid leukemia can activate FOXO3a by reducing AKT activity.^26^ According to Yang and Hung, the antitumor activity of FOXO3a can sensitize resistant tumor cells to radiotherapy through combined treatment of radiation with chemotherapy.^27^

A comparison of FOXO3a expression between MM and tumors pointed out that most samples of MM showed weak or no protein expression. These results suggest that, for mesenchymal tissues, an increment in FOXO3a expression might indicate the malignant potential of these tumors. The fact that a predicted tumor suppressor marker exhibits a higher expression in ULM and LMS caught the authors’ attention. In this sense, the authors found research showing that, in addition to its function as a tumor suppressor, nuclear FOXO3 can also promote tumor cell survival. In Chronic Myeloid Leukemia (CML), the inhibition or blockade of TGFβ-FOXO3a led to a significant reduction in the leukemia-initiating cell population.^28^ The authors showed that a combination of TGF-β inhibition, FOXO3a deficiency, and Imatinib treatment induces efficient depletion of CML *in vivo*. Another work showed a new mechanism contributing to multidrug resistance involving FOXO3a as a sensor for cytotoxic stress induced by anticancer therapies. It was observed that the sustained activation of FOXO3a promotes cell resistance and survival via activation of ABCB1 expression.^29^ In fibroblasts, similar to breast cancer cells, FOXO3a inhibited HIF1-induced apoptosis via CITED2, resulting in reduced expression of NAP1 and RTP801 (pro-apoptotic). Thus, FOXO3a plays an important role in the survival response of normal and cancer cells in response to hypoxic stress.^30^ In glioblastomas, it was observed that upregulation of FOXO3a is associated with tumor progression and worse prognosis for the patients. The authors proposed that FOXO3a may represent a new biomarker for prognosis or a potential therapeutic target in glioblastoma.^31^

Based on all this information, the authors attempted to understand the role and regulation of FOXO3a expression in the USMT. When the PI3K pathway is activated in response to growth factors, inhibition of FOXO3a activity occurs, preventing its translocation to the cell nucleus. Generally, FOXO3a loss of function occurs after its phosphorylation and consequent degradation.^1^,^4^ Several studies have shown the relevance of the HER-2/FOXO3a signaling pathway since these markers are associated with growth, proliferation, and cell survival. According to previous literature, overexpression of HER-2 due to gene amplification occurs in breast cancer, ovarian tumors, lung, colon, stomach, esophagus, endometrium, and cervix.^32^ In this series, no positive HER2 expression was found by immunohistochemistry. FISH (TMA samples) and SISH (whole tumor) analysis were performed to assess if this protein lack was a consequence of gene deletion, but no alteration was observed. These results were not surprising because Layfield and colleagues had studied HER-2 protein expression in the LMS and found only 20% of positive cases (4 out of 20 samples).^33^ Amant et al. demonstrated the absence of expression in uterine LMS, AS and SEE.^34^ The authors were not able to detect EGFR1 expression in those samples too.

In parallel, tumor samples were evaluated for two growth factors protein expression (EGF and VEGF – as ligand and signaling initiators). Among the main growth factors involved in the development of cancer which represent important therapeutic targets, are the Epidermal Growth Factor (EGF) and the Vascular Endothelial Growth Factor (VEGF). EGF acts on several cell types, including epithelial and non-epithelial cells. Here, cytoplasmic and nuclear EGF expression was observed, with a predominance of protein in the cytoplasm of malignant tumors. ULM sample showed a higher amount of the cytoplasmic protein expression followed by LMS, but no statistical significance was observed. Concerning nuclear expression of the EGF, apparently, the cell nucleus would be a second site of action of EGF and its receptor, and that location would be linked to the regulation of cell proliferation.^35^ Nuclear expression was found in LM samples, followed by ULM. The biological actions of EGF are mediated through its binding to its receptor and once activated, the receptors trigger the recruitment and phosphorylation of various intracellular substrates, leading to mitogenic signaling and other cellular activities. Moreover, VEGF pathway is well described as playing a role in tumoral angiogenesis and several therapeutic or neutralizing antibodies for the protein or its receptor have been documented.^36^ The present samples showed lower expression of VEGF with score values below the cut-off with detected protein only in ULM and LMS samples. Although a relevant role of IGF in the FOXO3a inactivation had been described,^37^ the authors did not perform this analysis, but a preliminary study from the group found that IGFR and ISR1 genes are upregulated in the LMS cell line.

To assess the prognostic role of FOXO3a expression, the clinical and pathological data of the LMS patients were evaluated in function of the wild type and phosphorylated form of the protein expression scores. Only metastasis occurrence showed significant association with phosphorylated protein expression. Relapse occurrence and distant metastasis were more frequent in those patients too, but without statistical significance (p>0.05). FOXO3a expression was associated with higher DFS in LMS patients, with no significant differences in OS. Yu and collaborators observed that patients with gastric cancer, showing a lack of FOXO3a expression, presented significantly lower OS than those patients with higher protein amounts.^38^ However, patients with increased FOXO3a expression, in the normal tissue cell nucleus adjacent to the tumor, had significantly lower OS than other patients. No significant differences were observed comparing the OS of patients with FOXO3a wild type or phosphorylated form. The authors hypothesize that the absence of statistical significance in the prognosis analyses was due to both the higher number of LMS positive samples, and because the overall and DFS are always lower in these patients too.

Regarding miRNA expression findings, it was observed that many of them presented high values of fold regulation, considering the overexpression or down expression values of ±2. Since all the miRNAs included in the plates had been previously described as being involved in carcinogenesis, their expression was expected to be lower in normal and in benign tissue. Among the six miRNAs (miR-96-5p, miR-222-3p, miR-132-3p, let7c-5p, miR-155-5p, and miR-25-3p) found with differential expression in the LMS samples, and that were described in the literature as FOXO3a regulators, miR-96-5p was the highest expressed one, followed by miR-155-5p. MiR-96-5p was found overexpressed both in tumors and serum from patients with ovarian cancer, which is a relevant hormone-dependent gynecologic cancer.^39^ In breastcancer cells and tissues, miR-96 was described as upregulated compared with the normal ones.^8^ This upregulation resulted in modulation of the cells' entry into the G1/S phase, as a consequence of the Cyclin-Dependent Kinase (CDK) inhibitors, p27 and p21 downregulation, and cyclin D1 upregulation. The authors demonstrated that miR-96 downregulates FOXO3a expression by targeting directly its 3′-untranslated region.^8^ In colorectal cancer, miR-96 seems to contribute to cell growth, and target directly TP53INP1, FOXO1 and FOXO3a53. These results indicate its potential to be used in miRNA-based therapies for patients. Concerning miR-155, Li and colleagues showed that its deficiency decreases vascular calcification due to increased Akt phosphorylation and FOXO3a degradation.^40^ Other study confirmed that miR-155 drives the angiogenesis in gastric cancer, enhancing the generation of new vessels *in vitro* through FOXO3a protein inhibition. The authors pointed to miR-155 as a potential biomarker for the detection of migration and angiogenesis in gastric cancer cells, suggesting that it could acts as a novel target for anti-angiogenesis therapy.^41^

**Figure 7:**
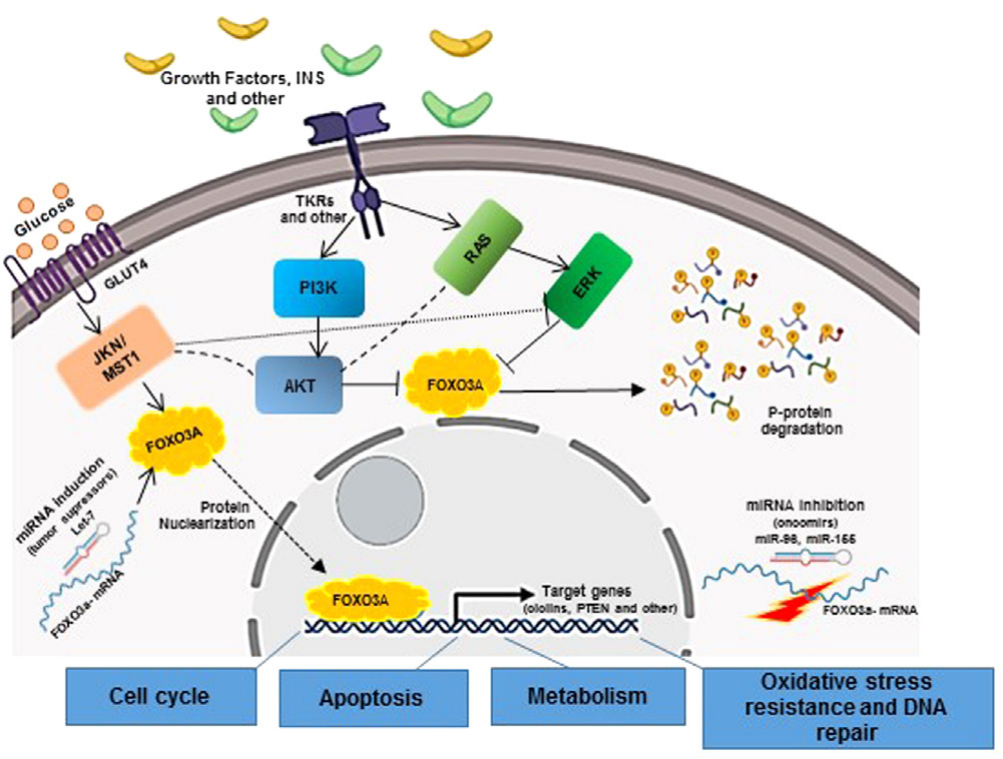
Potential mechanisms involved in FOXO3a regulation in the USMT. TKRs binding to growth factors that lead to AKT activity on FOXO3a is the mainly and well described mechanisms of FOXO3a inactivation by phosphorylation. In USMT tumors, several evidence have shown a relevant role of the IGF/IGFR signaling. MiRNAs have an increasing number of evidence both in USMT development as well as in FOXO3a regulation.

Overall, the present results, together with currently available data, show FOXO transcription factors as molecules that may present flexible action. They can have a suppressor or oncogenic role according to the timing of the cells and tissues. Additionally, FOXOs suppression in cancer cells is thought a consequence of multiple onco-kinase activation by a phosphorylation-ubiquitylation-mediated cascade. Therefore, several evidence show that inhibition of FOXO proteins would naturally occur due to a multifactorial post-translational process, as the authors hypothesize that occurs in USMT patients (Fig. 7).

To the best of our knowledge, this study is the first evidence of the FOXO3a expression in USMT. The results suggest the FOXO3a impairment is associated with these tumors' malignancy risk. These findings will contribute significantly to the biological knowledge of the USMT and, in the future, FOXO3a might become a potential prognostic marker to these patients. Beyond that, these molecules may represent potential therapeutic targets for individualized treatments for LMS patients. It is known that LMS precise diagnosis is only possible after surgery, so, the majority of the studies include a small number of samples. Here, the authors believe that this sample set has a size enough to make the present data robust. On the other side, the inclusion of some older specimens may limit the evaluation of some clinical data; but that does not invalidate the contribution of these findings to the knowledge of tumor biology.

## Authors' contributions

T.G.A performed all patients’ data and samples collection and performed the growth factors analysis. A.R.R performed the IHQ reactions and analysis for FOXO3a nuclear and cytoplasmic expression, and statistical analysis. L.G.A. performed miRNA analysis and manuscript review. G.A.R.M. and E.C.B contributed with intellectual support and manuscript review. K.C.C. idea conception, data analysis and manuscript preparation. All authors have read and agreed to the published version of the manuscript.

## Funding

This work was supported by the FAPESP grant – 2012/23652-0.

## Declaration of competing interest

The authors declare no conflict of interest and funding disclosures.

## Appendix Supplementary materials

Image, application 1
