# Supplementary material for: FOXO3a deregulation in uterine smooth muscle tumors
Source: Clinics (Sao Paulo). 2024 Apr 17;79:100350. doi: 10.1016/j.clinsp.2024.100350 (PMC11031728; doi:10.1016/j.clinsp.2024.100350)
Supplement: Supplementary file 1 [file mmc1.docx]

**CLINICS-D-23-00495_SUPPLEMENTARY MATERIAL**

**Table S1** Clinical features of the LM patients.

| **Characteristics** | **Categories** | **Number (%)** |
| --- | --- | --- |
| Ethnicity | Caucasian | 47 (59) |
|  | No-caucasian | 33 (44) |
| Menopause | Yes | 0 (0) |
|  | No | 100 (100) |
| Body Mass Index (Kg/m^2^)^a^ |  | 28 |
| Menarche Age (years) |  | 13 |
| Pregnancy | Yes | 50 (63) |
|  | No | 30 (37) |
| Smokers | Yes | 31 (38) |
|  | No | 49 (62) |
| Associated pathologies | Yes | 38 (48) |
| Hysterectomy | Yes | 62 (77) |
| Uterine volume (cm^3^)^b^ |  | 348 (±561) |
| Nodules (number) |  | 3 (±5,2) |
| Oral contraceptive | Yes | 49 (62) |
|  | No | 31 (38) |
| Treatment | Yes | 30 (37) |
|  | No | 50 (63) |

^a^ Kg/m^2^ – Kilogram/square meter.

^b^ cm^3^ – cubic centimeters.

**Table S2** List of genes included in the open array chip to assess the samples gene expression profile.

| **Gene symbol** | **Gene name** | ***TaqMan*^®^ assay ID** | **Reference** |
| --- | --- | --- | --- |
| ACTB* | Actin, beta | hs 99999903_m1 | NM_001101 |
| B2M* | Beta-2-microglobulin | hs 00984230_m1 | NM_004048 |
| GAPDH* | Glyceraldehyde-3-phosphate dehydrogenase | hs 99999905_m1 | NM_002046 |
| GUSB* | Glucuronidase, beta | hs 00939627_m1 | NM_000181 |
| HPRT1* | Hypoxanthine phosphoribosyltransferase 1 | hs 02800695_m1 | NM_000194 |
| RPLP0* | Ribosomal protein, large, P0 | hs 99999902_m1 | NM_001002 |
| APC | Adenomatous polyposis coli | hs 01568269_m1 | NM_000038 |
| AXIN1 | Axin 1 | hs 00394718_m1 | NM_003502 |
| AXIN2 | Axin 2 | hs 00610344_m1 | NM_004655 |
| BCL2 | B-cell CLL/lymphoma 2 | hs 00608023_m1 | NM_000633 |
| BMP2 | Bone morphogenetic protein 2 | hs 00154192_m1 | NM_001200 |
| BMP4 | Bone morphogenetic protein 4 | hs 01041266_m1 | NM_130851 |
| BMP5 | Bone morphogenetic protein 5 | hs 00234930_m1 | NM_021073 |
| BMP6 | Bone morphogenetic protein 6 | hs 01099594_m1 | NM_001718 |
| BMP7 | Bone morphogenetic protein 7 | hs 00233476_m1 | NM_001719 |
| BMP8B | Bone morphogenetic protein 8b | hs 01629120_s1 | NM_001720 |
| CCND1 | Cyclin D1 | hs 00765553_m1 | NM_053056 |
| CCND2 | Cyclin D2 | hs 00153380_m1 | NM_001759 |
| CSNK1A1 | Casein kinase 1, alpha 1 | hs 00793391_m1 | NM_001892 |
| CSNK2A1 | Casein kinase 2, alpha 1 polypeptide | hs 00953536_m1 | NM_001895 |
| CTNNB1 | Catenin (cadherin-associated protein), beta 1, 88kDa | hs 00355049_m1 | NM_001904 |
| CTNNBIP1 | Catenin, beta interacting protein 1 | hs 00172016_m1 | NM_020248 |
| DAAM1 | Dishevelled associated activator of morphogenesis 1 | hs 00323674_m1 | NM_014992 |
| DHH | Desert hedgehog | hs 00368306_m1 | NM_021044 |
| DISP1 | Dispatched homolog 1 (Drosophila) | hs 00399179_m1 | NM_032890 |
| DISP2 | Dispatched homolog 2 (Drosophila) | hs 00394338_m1 | NM_033510 |
| DVL1 | Dishevelled, dsh homolog 1 (Drosophila) | hs 00182896_m1 | NM_004421 |
| DVL2 | Dishevelled, dsh homolog 2 (Drosophila) | hs 00182901_m1 | NM_004422 |
| EIF5A | eucariotic initiation factor 5A | hs 04188519_m1 | NM_001143 |
| ERBB4 | V-erb-a erythroblastic leukemia viral oncogene homolog 4 | hs 00955525_m1 | NM_005235 |
| ESR1 | Estrogen receptor | hs 00174860_m1 | NM_001253 |
| FGF4 | Fibroblast growth factor 4 | hs 00999691_m1 | NM_002007 |
| FGF9 | Fibroblast growth factor 9 (GLIa-activating factor) | hs 00181829_m1 | NM_002010 |
| FGFR3 | Fibroblast growth factor receptor 3 | hs 00179829_m1 | NM_000142 |
| FOXE1 | Forkhead box E1 (thyroid transcription factor 2) | hs 00916085_s1 | NM_004473 |
| FOXN1 | Forkhead box N1 | hs 00186096_m1 | NM_003593 |
| FOXO3A | Forkhead box | hs 00818121_m1 | NM_001455 |
| FRZB | Frizzled-related protein | hs 00173503_m1 | NM_001463 |
| FZD1 | Frizzled family receptor 1 | hs 00268943_s1 | NM_003505 |
| FZD2 | Frizzled family receptor 2 | hs 00361432_s1 | NM_001466 |
| FZD4 | Frizzled family receptor 4 | hs 00201853_m1 | NM_012193 |
| FZD5 | Frizzled family receptor 5 | hs 00258278_s1 | NM_003468 |
| FZD6 | Frizzled family receptor 6 | hs 00171574_m1 | NM_003506 |
| FZD7 | Frizzled family receptor 7 | hs 00275833_s1 | NM_003507 |
| FZD8 | Frizzled family receptor 8 | hs 00259040_s1 | NM_031866 |
| FZD9 | Frizzled family receptor 9 | hs 00268952_s1 | NM_003508 |
| GAS1 | Growth arrest-specific 1 | hs 00266715_s1 | NM_002048 |
| GLI1 | GLI family zinc finger 1 | hs 01110766_m1 | NM_005269 |
| GLI2 | GLI family zinc finger 2 | hs 01119974_m1 | NM_005270 |
| GLI3 | GLI family zinc finger 3 | hs 00609233_m1 | NM_000168 |
| GREM1 | Gremlin 1 | hs 00171951_m1 | NM_013372 |
| GSK3A | Glycogen synthase kinase 3 alpha | hs 00997938_m1 | NM_019884 |
| GSK3B | Glycogen synthase kinase 3 beta | hs 01047719_m1 | NM_002093 |
| HHAT | Hedgehog acyltransferase | hs 00911326_m1 | NM_018194 |
| HHIP | Hedgehog interacting protein | hs 01011015_m1 | NM_022475 |
| IFT52 | Intraflagellar transport 52 homolog (Chlamydomonas) | hs 00211198_m1 | NM_016004 |
| IHH | Indian hedgehog | hs 01081801_m1 | NM_002181 |
| JUN | Jun proto-oncogene | hs 01103582_s1 | NM_002228 |
| LATS1 | LATS, large tumor suppressor, homolog 1 (Drosophila) | hs 01125528_m1 | NM_004690 |
| LATS2 | LATS, large tumor suppressor, homolog 2 (Drosophila) | hs 00324396_m1 | NM_014572 |
| LEF1 | Lymphoid enhancer-binding factor 1 | hs 01547250_m1 | NM_016269 |
| MAPK1 | Mitogen-activated protein kinase 1 | hs 01046830_m1 | NM_002745 |
| MMP7 | Matrix metallopeptidase 7 (matrilysin, uterine) | hs 01042796_m1 | NM_002423 |
| MAPK8 | Mitogen-activated protein kinase 8 | hs 00177083_m1 | NM_002750 |
| MTSS1 | Metastasis suppressor 1 | hs 00207341_m1 | NM_014751 |
| MYC | V-myc myelocytomatosis viral oncogene homolog (avian) | hs 00153408_m1 | NM_002467 |
| PGR | progesterone receptor | hs 01556702_m1 | NM_000926 |
| PRL | prolaction | hs 00168730_m1 | NM_000948 |
| PRLR | prolactin receptor | hs 01061477_m1 | NM_000949 |
| PTCH1 | Patched 1 | hs 00181117_m1 | NM_000264 |
| PTCH2 | Patched 2 | hs 00184804_m1 | NM_003738 |
| PTCHD1 | Patched domain containing 1 | hs 00288486_m1 | NM_173495 |
| PTCHD2 | Patched domain containing 2 | hs 01367724_m1 | NM_020780 |
| PTCHD3 | Patched domain containing 3 | hs 01584645_m1 | NM_001034 |
| PTEN | protein tensin homologue | hs 02621230_s1 | NM_000314 |
| RAB23 | RAB23, member RAS oncogene family | hs 00212407_m1 | NM_183227 |
| RHOA | Ras homolog gene family, member A | hs 00357608_m1 | NM_001664 |
| SFRP1 | Secreted frizzled-related protein 1 | hs 00610060_m1 | NM_003012 |
| SFRP4 | Secreted frizzled-related protein 4 | hs 00180066_m1 | NM_003014 |
| SHH | Sonic hedgehog | hs 00179843_m1 | NM_000193 |
| SLC2A1 | Facilitated glucose transporter member 1 | hs 00892681_m1 | NM_001455 |
| SLC2A3 | Facilitated glucose transporter member 3 | hs 00359840_m1 | NM_006931 |
| SLC2A4 | Facilitated glucose transporter member 4 | hs 00168966_m1 | NM_001042 |
| SMO | Smoothened, frizzled family receptor | hs 01090242_m1 | NM_005631 |
| SUFU | Suppressor of fused homolog (Drosophila) | hs 00171981_m1 | NM_016169 |
| TCF7 | Transcription factor 7 (T-cell specific, HMG-box) | hs 00175273_m1 | NM_003202 |
| TCF7L1 | Transcription factor 7-like 1 (T-cell specific, HMG-box) | hs 01064103_m1 | NM_031283 |
| TLE1 | Transducin-like enhancer of split 1 (E(sp1) homolog, Drosophila) | hs 00270768_m1 | NM_005077 |
| TP53 | Tumor protein p53 | hs 01034249_m1 | NM_000546 |
| VEGFA | Vascular endothelial growth factor A | hs 00900055_m1 | NM_003376 |
| WIF1 | WNT inhibitory factor 1 | hs 00183662_m1 | NM_007191 |
| WISP1 | WNT1 inducible signaling pathway protein 1 | hs 04234730_m1 | NM_003882 |
| WNT1 | Wingless-type MMTV integration site family, member 1 | hs 01011247_m1 | NM_005430 |
| WNT10A | Wingless-type MMTV integration site family, member 10A | hs 00228741_m1 | NM_025216 |
| WNT10B | Wingless-type MMTV integration site family, member 10B | hs 00559664_m1 | NM_003394 |
| WNT11 | Wingless-type MMTV integration site family, member 11 | hs 00182986_m1 | NM_004626 |
| WNT16 | Wingless-type MMTV integration site family, member 16 | hs 00365138_m1 | NM_057168 |
| WNT2 | Wingless-type MMTV integration site family member 2 | hs 00608224_m1 | NM_003391 |
| WNT2B | Wingless-type MMTV integration site family, member 2B | hs 00921614_m1 | NM_004185 |
| WNT3 | Wingless-type MMTV integration site family, member 3 | hs 00902257_m1 | NM_030753 |
| WNT3A | Wingless-type MMTV integration site family, member 3A | hs 00263977_m1 | NM_033131 |
| WNT4 | Wingless-type MMTV integration site family, member 4 | hs 01573504_m1 | NM_030761 |
| WNT5A | Wingless-type MMTV integration site family, member 5A | hs 00998537_m1 | NM_003392 |
| WNT5B | Wingless-type MMTV integration site family, member 5B | hs 01086864_m1 | NM_032642 |
| WNT6 | Wingless-type MMTV integration site family, member 6 | hs 00362452_m1 | NM_006522 |
| WNT7A | Wingless-type MMTV integration site family, member 7A | hs 01114990_m1 | NM_004625 |
| WNT7B | Wingless-type MMTV integration site family, member 7B | hs 00536497_m1 | NM_058238 |
| WNT8A | Wingless-type MMTV integration site family, member 8A | hs 00230534_m1 | NM_058244 |
| WNT8B | Wingless-type MMTV integration site family, member 8B | hs 00610126_m1 | NM_003393 |
| WNT9A | Wingless-type MMTV integration site family, member 9A | hs 00243321_m1 | NM_003395 |
| WNT9B | Wingless-type MMTV integration site family, member 9B | hs 00287409_m1 | NM_003396 |

**Figure S1** Representative Hematoxylin-Eosin (HE) stained samples of Leiomyoma (LM), Uncommon Leiomyoma (ULM) and Leiomyosarcoma (LMS). HER2 positive control for Iimmunohistochemical (IHC), FISH and CISH are presented in the lower panel.


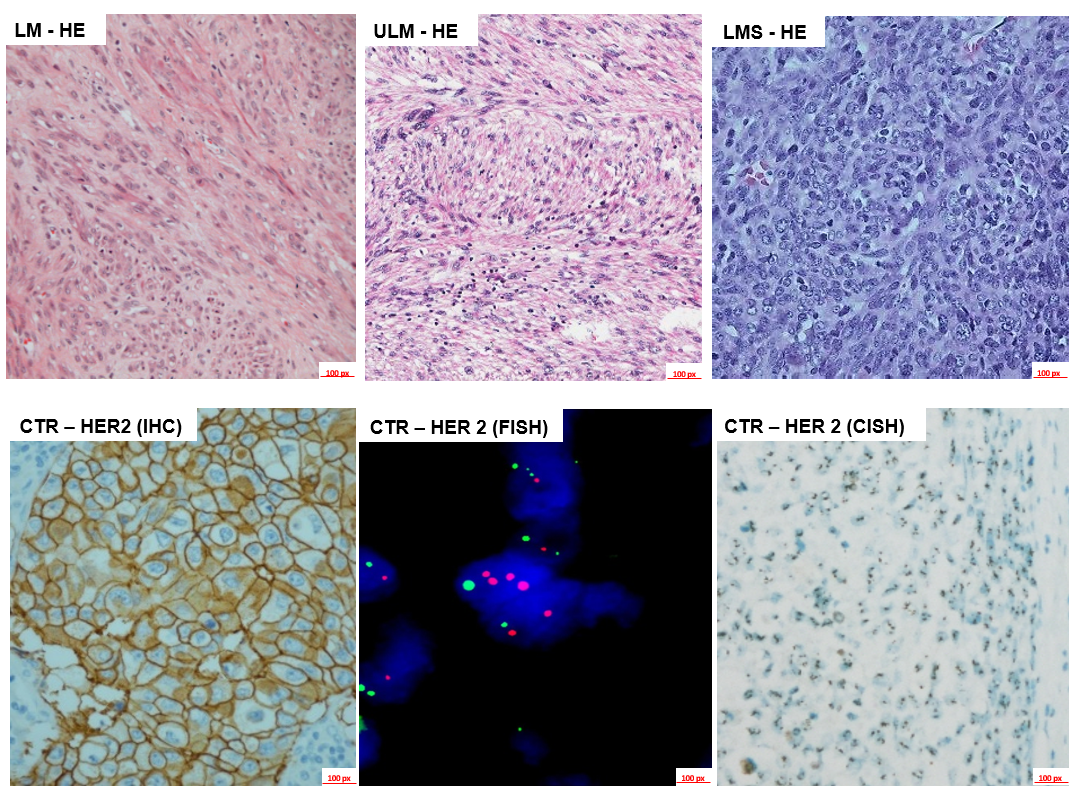


**Figure S2** Wild type and phosphorylated FOXO3a proteins. (A) Antibodies binding sites (green – phosphorylated, yellow ‒ wild type protein). (B) Western blot membrane showing the pattern of FOXO3a and p-FOXO3a detection using a total extract of MCF7 cells (breast cancer). The molecular weights of the proteins are indicated. Ct+ ‒ actin (1:2000, Thermo Scientific), Cytoplasm (1:500, Arigobio) and Nuclear (1:500, Novus Biological Inc) antibodies.

**
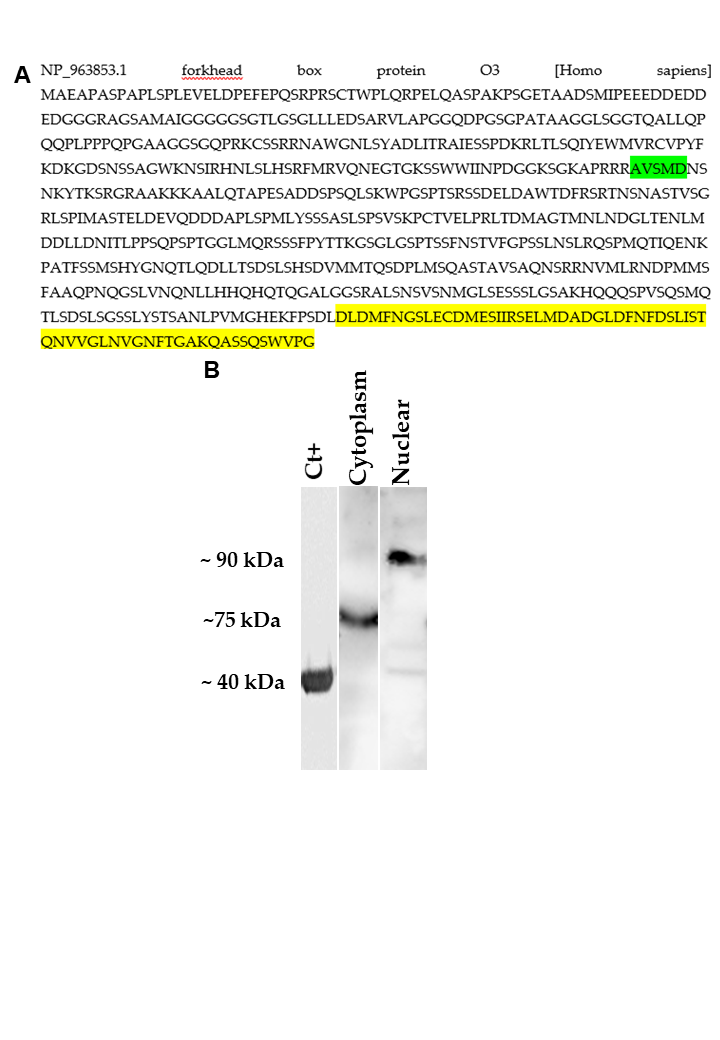
**
